# Real-world cost-effectiveness of denosumab for the treatment of postmenopausal osteoporosis in Taiwan

**DOI:** 10.1007/s11657-021-01020-6

**Published:** 2021-10-12

**Authors:** Ben Johnson, Edward Chia-Cheng Lai, Huang-tz Ou, Hong Li, Björn Stollenwerk

**Affiliations:** 1 Amgen Ltd, Uxbridge, UK; 2 Institute of Clinical Pharmacy and Pharmaceutical Sciences, College of Medicine, National Cheng Kung University, Tainan, Taiwan; 3 Amgen Asia Holding Limited, Quarry Bay, Hong Kong; 4 Amgen GmbH, Rotkreuz, Switzerland

**Keywords:** Denosumab, Real-world, Cost-effectiveness, Osteoporosis, Postmenopausal, Taiwan

## Abstract

**Summary:**

This study assessed the cost-effectiveness of continued denosumab treatment, compared with discontinuation of denosumab after one dose, for the treatment of postmenopausal osteoporosis in Taiwan, using real-world fracture reduction effectiveness and cost data. Outcomes indicate that continued denosumab treatment produces an incremental cost-effectiveness ratio of USD $16,743 per QALY.

**Purpose:**

To evaluate the cost-effectiveness of continued denosumab use versus discontinuation after one dose, for the treatment of postmenopausal osteoporosis in Taiwan, using real-world fracture reduction effectiveness and cost data.

**Methods:**

A Markov cohort model was used to evaluate the lifetime costs and QALYs associated with continued denosumab treatment versus discontinuation of treatment after one dose. The evaluation was conducted from the perspective of Taiwan’s healthcare system and used a discount rate of 3% per annum. The patient population consisted of postmenopausal women with osteoporosis with a mean age of 77 years who initiated denosumab treatment. Fracture reduction effectiveness data, baseline fracture rates, mortality data, and costs of fracture were informed by Taiwan’s National Health Insurance Research Database.

**Results:**

Model outcomes showed that continued treatment with denosumab produced an expected gain of 0.042 QALYs and an incremental cost of USD $704, compared with discontinuation of denosumab after one dose. This corresponds to an incremental cost-effectiveness ratio of USD $16,743 per QALY gained. Probabilistic and scenario analysis showed that results are stable to variations in model assumptions and parameters.

**Conclusion:**

In a real-world setting, at a cost per QALY threshold equivalent to gross domestic product per capita in 2020 in Taiwan (USD $30,038), continued treatment with denosumab in postmenopausal women with osteoporosis is cost-effective compared with treatment discontinuation.

## Introduction

Osteoporosis is a common condition, whose prevalence is increasing with the progressively aging population in Asia. Fragility fractures are the main consequence of osteoporosis, which, as well as resulting in increased mortality and diminished health-related quality of life (HRQoL), also produce a substantial economic burden [1].

Denosumab is a fully human monoclonal antibody that targets and inhibits RANK-L, a ligand which stimulates bone resorption, with high specificity. Denosumab is currently approved as a treatment for postmenopausal osteoporosis in more than 80 healthcare markets globally [2–5], including in Taiwan, where it has been marketed since 2011 [6], and is one of the most frequently used antiresorptive treatments under Taiwan’s National Health Insurance system [7]. As with most pharmacological treatments, compliance with osteoporosis therapies has been shown to be an important factor in achieving treatment success [8].

During the past decade, several economic models have been published evaluating the cost-effectiveness of denosumab compared with either no treatment or other pharmacological therapies [9–12]. All these studies used efficacy data from randomized clinical trials (RCT), or network meta-analyses of RCTs, to inform the fracture reduction effects of pharmacological therapy, thereby making the assumption that the treatment efficacy observed in trials translates to clinical practice. To address this potential limitation, the cost-effectiveness of denosumab was assessed using treatment effectiveness data from a robust real-world study.

A retrospective database study evaluating the effectiveness and safety of denosumab in clinical practice among women with postmenopausal osteoporosis in Taiwan and Hong Kong using National Health Insurance claims data was completed in 2019 [13], referred to the Taiwan Real-World Study (TWRWS) hereinafter. Employing the propensity score (PS) matching approach, this study compared the real-world clinical fracture incidence in patients who received at least 2 doses of denosumab (referred to as the “treatment cohort”) and patients who discontinued after 1 dose of denosumab (referred to as “off-treatment cohort”) between 2012 and 2016, using an index date of 225 days after the initial dose. The off-treatment cohort was selected to minimize confounding relating to the initial treatment decision, and because this patient group can be considered a proxy for a “no treatment” cohort, given that any fracture reduction benefit from the initial denosumab dose is unlikely to persist beyond the study index date. PS matching was undertaken based on 59 baseline variables, all of which were balanced between cohorts following the matching process, defined as exhibiting a standardized mean difference < 0.1. Results of the study showed that the risk of hip fracture, clinical vertebral fracture, and non-vertebral fracture was reduced in the Taiwanese treatment cohort by 38% (hazard ratio, 0.62 [95% CI: 0.52, 0.75]), 37% (hazard ratio, 0.63 [95% CI: 0.52, 0.75]), and 38% (hazard ratio, 0.62 [95% CI: 0.53, 0.73), respectively.

The objective of this economic analysis is to evaluate the cost-effectiveness of continued treatment with denosumab versus discontinuation of denosumab after one dose in postmenopausal women with osteoporosis in Taiwan, using fracture reduction effectiveness outcomes from the TWRWS. The study design was selected to align with the design of the TWRWS as closely as possible, and therefore assessed the cost-effectiveness of persistence with denosumab versus non-persistence, rather than the cost-effectiveness of treatment versus no treatment per se. However, given that patients in the “off-treatment cohort” received only a single dose of denosumab, outcomes are also likely to represent a reasonable proxy for the latter comparison. To the best of the authors’ knowledge, this study is the first to use denosumab treatment effectiveness data from a real-world study to inform an economic analysis.

## Methods

### Model overview

A cost-effectiveness model, whose structure has been described previously [9, 10, 14], was adapted to evaluate lifetime costs and quality-adjusted life years (QALYs) associated with continued denosumab use (treatment cohort) and discontinuation of denosumab after one dose (off-treatment cohort). The analysis was conducted from the perspective of Taiwan’s healthcare system and used an annual discount rate of 3% for costs and health outcomes, in line with local guidelines [15]. Costs were assessed in 2020 US dollars.

**Fig. 1:**
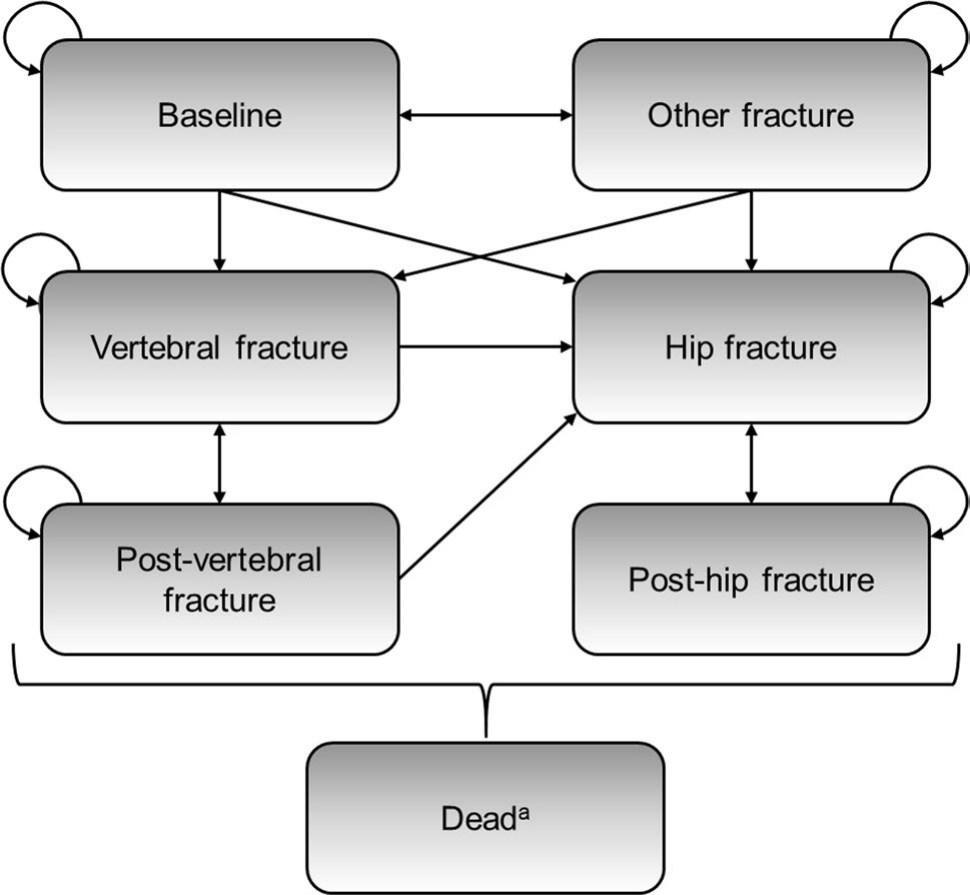
Structure of the model. ^a^Death can occur from any other Markov state. Transitions are not shown for simplicity

The model used a Markov cohort structure (shown in Fig. 1) to assess the incidence of hip, vertebral, and “other” (wrist or distal forearm) fractures over time. All patients start in the “well” state, and in each 6-month model cycle are at risk of experiencing a fragility fracture (hip, vertebral, or “other” fracture) or dying. To track the long-term impact of fractures on health-related quality of life (HRQoL), healthcare costs, and mortality, after 1 year in the hip fracture or vertebral fracture state, patients transition to the post-hip fracture and post-vertebral fracture states, respectively. After 1 year in the “other” fracture state, patients transition back to the well state.

The model uses a hierarchical structure, based on the severity of fracture types, with hip fracture being the most severe, and “other” fracture the least severe. Patients in hip fracture states cannot sustain a subsequent vertebral or “other” fracture, and patients in the vertebral fracture states cannot sustain an “other” fracture. To adjust for the underestimation of vertebral and “other” fracture incidence imposed by this structure, a correction factor was implemented, where the incidence of overlooked fractures and their impact on costs, HRQoL, and mortality was estimated independently of the Markov process, as described previously [14].

### Patient population

The modelled population was based on participants in the TWRWS [13]: postmenopausal women with osteoporosis who initiated treatment with denosumab, with a mean age of 77 years. The model simulated patients from the time at which the first dose of denosumab was administered.

### Treatment duration

Patients in the off-treatment cohort received a single dose of denosumab, covering a 6-month period. Patients in the treatment cohort were assumed to receive denosumab treatment for an intended duration of 5 years, consistent with previous economic analyses [9, 10, 14].

Following the second dose of denosumab in the treatment cohort, treatment persistence was modelled using a previously published modelling framework [16]. In each cycle, a proportion of patients discontinued treatment, based on a discontinuation rate of 0.317 per patient-year among patients in the treatment cohort of the TWRWS, which was converted to a discontinuation probability of 14.7% per 6-month model cycle, assuming a constant rate over time.

Clinical evidence has shown that the fracture reduction benefit of osteoporosis treatment continues for some time after discontinuation, rather than stopping immediately [16]. Therefore, in line with previous peer-reviewed economic analyses of denosumab, the assumption was made that treatment benefit decreases linearly to 0 over a period of 2 years following discontinuation [10, 17].

### Clinical inputs

Fracture incidence in untreated patients was informed by annual rates of hip, vertebral, and “other” (wrist or distal forearm) fracture from the off-treatment cohort of the TWRWS [13] (non-PS matched). Fractures were identified using diagnosis codes from the International Classification of Diseases, 9th Revision, Clinical Modification until December 2015, and the International Classification of Diseases, 10th Revision, Clinical Modification thereafter [18], with validation of hip fractures conducted by external medical chart review. Fractures occurring on the same date as a motor vehicle accident were excluded, to increase the probability that fractures were due to osteoporosis.

To inform fracture rates in denosumab-treated patients, hazard ratios of fracture (PS matched) for the treatment versus off-treatment cohort of the TWRWS were applied to fracture rates in untreated patients. As with previous analyses, a hazard ratio for nonvertebral fracture was used to inform “other” fracture incidence [9, 10]. Hazard ratios were applied for persistent patients in the treatment cohort during the 5-year treatment course (and during the offset period after treatment discontinuation), and for the first cycle of the model for the off-treatment cohort, to account for the single dose of denosumab received by these patients. Fracture rates and treatment effectiveness data used in the model are shown in Table 1.

**Table 1:** Model input parameters

Input	Value (95% CIs)			Source
Fracture reduction efficacy	Hip fracture	Vertebral fracture	Nonvertebral fracture	
Hazard ratio—treatment vs off-treatment cohort	0.62 (0.52 to 0.75)	0.63 (0.52 to 0.75)	0.62 (0.53 to 0.73)	TWRWS [13]
Fracture rates in the off-treatment cohort	Hip fracture	Vertebral fracture	Other fracture	
Annual fracture rate	0.017 (0.016 to 0.019)	0.017 (0.015 to 0.018)	0.004 (0.003 to 0.005)	TWRWS [13]
Annual mortality in the “well” state	Value			
Age 70–79 years	0.041			Chang 2016 [19]
Age 80–89 years	0.089			Chang 2016 [19]
Age 90 +	0.168			Chang 2016 [19]
Relative risks of death following fracture	Hip fracture	Vertebral fracture	Other fracture	
First year after fracture—age 70–79 years	1.57	1.26	0.97	Chang 2016 [19]
First year after fracture—age 80–89 years	1.46	1.18	1.01	Chang 2016 [19]
First year after fracture—age 90 + years	1.5	1.15	1.72	Chang 2016 [19]
Second and subsequent years after fracture—age 70–79 years	1.69	1.27	-	Chang 2016 [19]
Second and subsequent years after fracture—age 80–89 years	1.37	1	-	Chang 2016 [19]
Second and subsequent years after fracture—age 90 + years	1.12	1.15	-	Chang 2016 [19]
Medical costs of fracture	Hip fracture	Vertebral fracture	Other fracture	
First year after fracture—age 70–79 years	$4,315	$1,604	$1,117	Chang 2016 [19]
First year after fracture—age 80–89 years	$4,395	$1,844	$1,571	Chang 2016 [19]
First year after fracture—age 90 + years	$3,930	$1,354	$1,281	Chang 2016 [19]
Second and subsequent years after fracture	$1,163	$734		Chang 2016 [19]
Drug costs and treatment management	Value			
Denosumab—annual drug cost	$400.00			Taiwan NHIA [22]
Routine healthcare visit	$10.54			Taiwan NHIA [23]
Nurse visit	$12.04			Taiwan NHIA [23]
Bone densitometry scan	$20.01			Taiwan NHIA [23]
General population health-related quality of life	Value			
Age 75–79 years	0.669			Sun 2008 [24]
Age 80–84 years	0.655			Sun 2008 [24]
Age 85 + years	0.643			Sun 2008 [24]
Utility multipliers	Hip fracture	Vertebral fracture	Other fracture	
1st year after fracture	0.55 (0.53 to 0.57)	0.68 (0.65 to 0.70)	0.83 (0.82 to 0.84)	Svedbom 2018 [25]
2nd and following years after fracture	0.86 (0.84 to 0.89)	0.85 (0.82 to 0.87)	-	Svedbom 2018 [25]

*CI*, confidence interval; *TWRWS*, Taiwan Real-World Study

### Mortality

Probabilities of death in the model were derived from Chang et al. (2016) [19]: a study comparing mortality and healthcare costs among postmenopausal women following osteoporotic fracture with age- and comorbidity-matched controls, using data from Taiwan’s National Health Insurance Research Database (NHIRD). Age-specific mortality for patients in the “well” state was informed by death rates in matched control groups, estimated as a weighted average across control groups for all fracture types. To account for the increased mortality risk due to fracture, age-specific relative risks of death for the case versus control cohort were applied in the first year after hip, vertebral, and “other” fracture, and for the second and following years after hip and vertebral fracture. Since the study did not explicitly report relative risks of death for “other” fractures, these values were estimated as a weighted average of mortality outcomes for wrist and upper end humerus fracture. Mortality inputs used in the model are shown in Table 1.

Consistent with previous denosumab economic analyses [9, 10], the model assumed that excess fracture-related mortality persists for 8 years after the event for hip and vertebral fracture, and 1 year after the event for “other” fracture.

### Costs

The model included two categories of cost: medical costs due to fracture and treatment costs. Costs were adjusted to 2020 values using inflation data for Taiwan [20], and converted from New Taiwan dollars to US dollars where required, using an exchange rate of 0.0334 USD per TWD [21].

Age-specific medical costs due to fracture were informed by Taiwan’s National Health Insurance Research Database (NHIRD) [19], and included a first-year cost for all fracture types (hip, vertebral, and “other”), and a recurring annual cost in the second and subsequent years after the event for hip and vertebral fracture. As with fracture-related mortality inputs, the first-year cost of “other” fractures was informed by a weighted average of the cost of wrist and upper end humerus fracture.

Treatment costs included the annual drug cost of denosumab (Prolia®), informed by Taiwan’s National Health Insurance Administration [22], and an annual treatment management cost, which consisted of a routine healthcare visit every year, a bone densitometry (DXA) scan every 2 years, and a nurse visit every 6 months for denosumab administration [23]. Cost inputs used in the model are shown in Table 1.

### Health-related quality of life

Age-specific general population EQ-5D scores for Chinese women [24] were used to inform health-related quality of life (HRQoL) for patients in the “well” state. To account for the HRQoL reduction due to fracture, utility multipliers were applied to “well” state utilities for the first year after hip, vertebral, and “other” fracture, and in the second and following years after hip and vertebral fracture. These values were taken from an analysis of data from the International Costs and Utilities Related to Osteoporotic Fractures Study (ICUROS) [25]. Inputs relating to HRQoL are shown in Table 1.

### Analysis

The model assessed outcomes in terms of total discounted lifetime costs and QALYs for each intervention. In the base case, results were produced using point estimates for each model input. The economic analysis used the annual nominal gross domestic product (GDP) per capita in Taiwan (USD $30,038 [26]) as a willingness-to-pay threshold per QALY gained: a conservative approach based on the commonly used range of one to three times national GDP per capita in countries without an explicit threshold [27].

Uncertainty in model outcomes was addressed through scenario analyses, which explored the impact of (1) assuming full treatment persistence in the treatment cohort and (2) adjusting fracture rates to account for the ageing of the cohort over time. This second scenario analysis was conducted because fracture rates typically increase as patients age. However, age-stratified fracture rates were not available from the Taiwan real-world study, so were assumed to remain constant over time in the base case. To explore this assumption, as a scenario analysis, external data on the relative incidence of fractures in Swedish women of different ages [28] were used to estimate changing fracture rates over time for the Taiwanese cohort.

Uncertainty was also assessed via probabilistic sensitivity analysis, where model parameters were simultaneously stochastically varied, according to probability distributions informed by their point estimates and standard errors (taken from publications where available or assumed to be 10% of the mean otherwise), for 1,000 model iterations. This allowed the probability that each intervention is cost-effective to be quantified over a range of willingness to pay thresholds.

## Results

Base case cost-effectiveness results (displayed in Table 2) show that continued treatment with denosumab for up to 5 years (the treatment cohort) produces a reduction in fractures compared with discontinuation of denosumab after one dose (the off-treatment cohort); a total of 45 fractures are avoided per 1,000 patients over a 10-year period. This fracture reduction translates into a lifetime gain of 0.023 life years and 0.042 QALYs per patient. Continued treatment with denosumab is associated with a higher drug cost and treatment management cost (increase of $994), which is partially offset by a $290 reduction in lifetime fracture costs, producing a total incremental cost of $704. Continued denosumab treatment therefore produces an incremental cost-effectiveness ratio (ICER) of $16,743 per QALY compared with discontinuation of denosumab after one dose.

**Table 2:** Base case cost-effectiveness results

Outcome	Treatment cohort	Off-treatment cohort	Difference
10-year fracture incidence per 1,000 patients			
Hip fractures	105	126	− 21
Vertebral fractures	101	120	− 19
Other fractures	25	30	− 5
Total fractures	231	276	− 45
Costs (USD in 2020; discounted)			
Fracture cost	$1,708	$1,999	− $290
Drug cost	$1,094	$200	$894
Treatment management cost	$122	$22	$100
Total cost	$2,925	$2,221	$704
QALYs and Life years (discounted)			
Life-years	8.456	8.432	0.023
QALYs	5.389	5.347	0.042
Cost per QALY gained (USD)	$16,743		

Scenario analysis results (displayed in Table 3) show that assuming 100% persistence among patients who continue denosumab treatment increases the ICER versus patients who discontinue after one dose to $20,882 per QALY. Contrastingly, adjusting fracture rates to account for increasing fracture risk as the cohort ages reduces the ICER to $14,452.

**Table 3:** Scenario analysis results—treatment cohort versus off-treatment cohort

Scenario	∆ Costs (USD)	∆ QALYs	ICER (USD)
Base case	$704	0.042	$16,743
Perfect persistence assumed in persistent cohort	$1,239	0.059	$20,882
Fracture rates adjusted for age	$672	0.047	$14,452

**Fig. 2:**
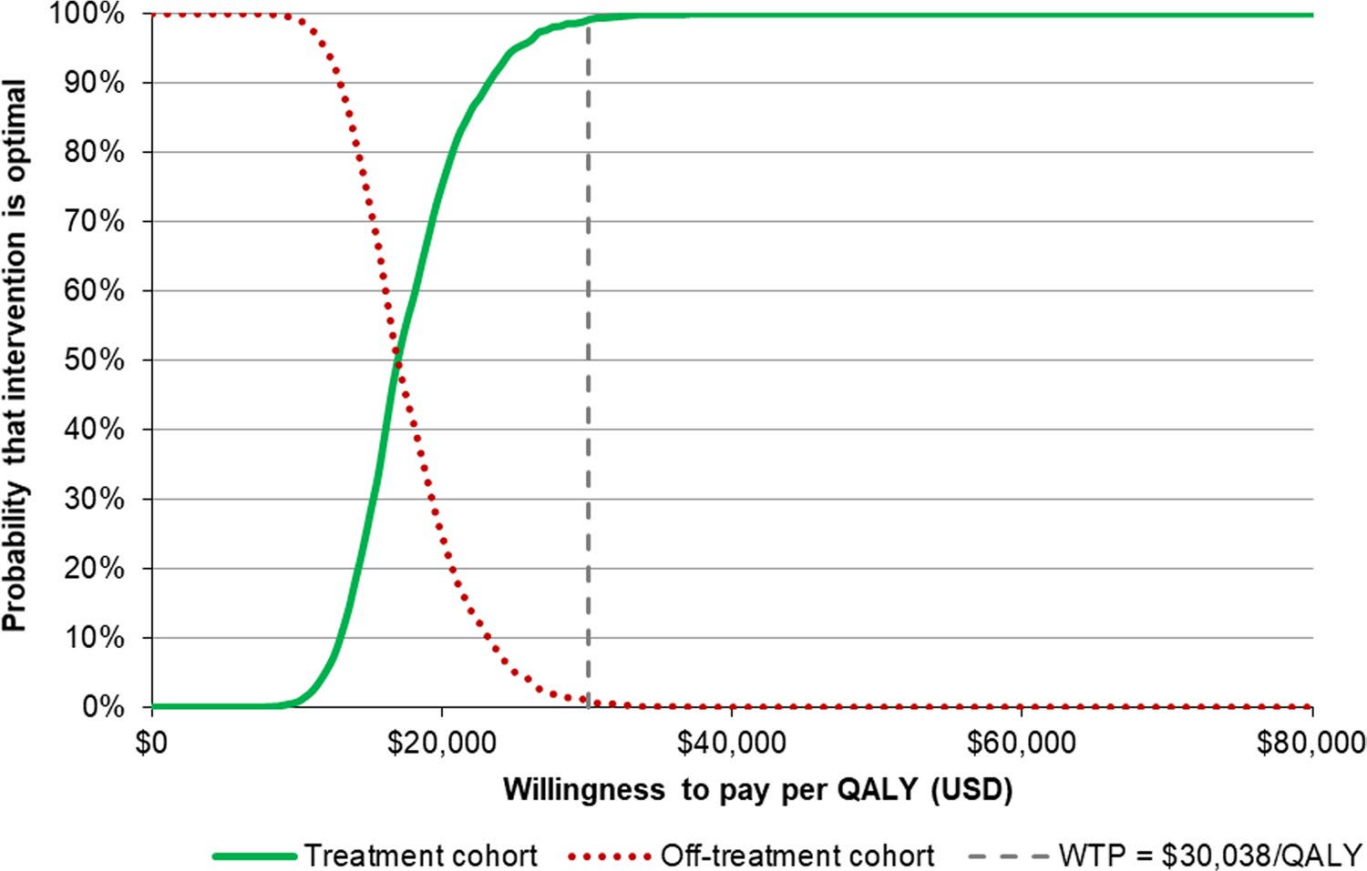
Cost-effectiveness acceptability curves—showing the number of iterations in which continued denosumab treatment (treatment) and discontinuation of denosumab after one dose (off-treatment) are cost-effective over a range of willingness to pay thresholds. QALY, quality-adjusted life year; USD, United States dollar; WTP, willingness to pay

Probabilistic sensitivity analysis results are displayed as cost-effectiveness acceptability curves in Fig. 2. These results show that continued treatment with denosumab is cost-effective in 99.0% of stochastic iterations at a cost per QALY threshold equivalent to Taiwan’s GDP per capita (USD $30,038 [26]). Continued denosumab treatment produces the highest number of QALYs in all stochastic iterations, reflected by the fact that this strategy becomes cost-effective in 100% of samples as the willingness to pay threshold rises.

## Discussion

This study assessed the cost-effectiveness of continued treatment with denosumab (the “treatment cohort”) with discontinuation of denosumab after one dose (the “off-treatment cohort”), using real-world effectiveness and cost data from Taiwan’s NHIRD. Results show that, compared with discontinuation after one dose, continued treatment with denosumab produces a gain of 0.042 QALYs, due to the fracture reduction benefit of treatment, and an incremental cost of USD $704 (despite a reduction in fracture costs), due to the higher drug cost. Continued denosumab treatment therefore produces an ICER of $16,743 per QALY, indicating that this strategy is cost-effective at a cost per QALY threshold equivalent to Taiwan’s GDP per capita (USD $30,038 [26]). As suggested by the World Health Organization [27], in settings without an explicit willingness-to-pay threshold, interventions with an ICER below three times local GDP per capita are commonly considered cost-effective, and those with an ICER below one times GDP per capita considered highly cost-effective. Scenario analysis and probabilistic sensitivity analysis show that cost-effectiveness results are generally stable to variation in model assumptions and parameters.

The modelling approach used in this study is generally consistent with previous work in the field. The model structure has been used to evaluate the cost-effectiveness of denosumab in various settings, including Sweden [9, 29], the USA [10], Canada [30], and Thailand [17]. Like a number of prior economic evaluations of osteoporosis therapies [31–33], this structure is based on the widely accepted International Osteoporosis Foundation reference model.

To the best of the authors’ knowledge, this study is distinct from previous economic evaluations of denosumab in two ways. First, model inputs were taken from a single real-world source (the NHIRD) wherever possible. In the current study, real-world *effectiveness* data were used to inform relative fracture incidence between arms. By contrast, previous economic analyses [9, 10, 17, 29, 30] used treatment *efficacy* data from RCTs, or network meta-analyses of RCTs, which predominantly relied on the FREEDOM trial [2] (the pivotal phase III denosumab RCT) for the fracture reduction benefit of denosumab. Furthermore, in the current study, fracture rates, treatment discontinuation data, fracture costs, and excess mortality were all informed by the NHIRD, whereas previous evaluations used data from a variety of sources. Using real-world data addresses issues with the generalizability of RCT evidence to clinical practice. For instance, real-world patients are likely to exhibit lower treatment compliance, and are likely to be more diverse in characteristics than participants in RCTs. Additionally, according to reimbursement guidance from Taiwan’s National Insurance Program, only patients with a prior fracture are eligible for osteoporosis treatment [34]. This is reflected in the substantially higher proportion of patients with a fracture at baseline in the TWRWS, compared with the FREEDOM trial [2].

A second way in which the current study differs from previous analyses is the choice of comparators. This comparison is consistent with the study design of the TWRWS, which assessed fracture outcomes in patients who received at least two doses of denosumab versus those who discontinued denosumab after one dose to minimize confounding; a real-world study comparing denosumab-treated patients with untreated patients would be subject to bias, since the two patient groups are likely to differ in terms of characteristics and risk factors. To align as closely with the available evidence as possible, the current evaluation modelled the two cohorts as they were defined in the TWRWS study, and therefore assessed the cost-effectiveness of persistence with denosumab, rather than using the “off-treatment” cohort to represent a “no treatment” arm. However, considering that the residual fracture reduction benefit from the initial dose of denosumab in the off-treatment TWRWS cohort is likely to be minimal, outcomes of this evaluation are also likely to represent a reasonable proxy for the real-world cost-effectiveness of denosumab versus no treatment.

As with all economic evaluations, this study has several limitations. First, while model parameters were sourced from NHIRD data wherever available, this was not possible in all cases. Of note, general population health-related quality of life (HRQoL) scores were informed by mainland Chinese data. Although the populations of mainland China and Taiwan are similar, healthcare systems, and potentially health state valuations, differ between the two locations. Therefore, further research is required to produce Taiwan-specific data to inform future economic analyses.

Second, the Taiwan real-world study did not include all types of fragility fracture; only hip, vertebral, humerus, and distal forearm fractures were considered. Although these are regarded as the most important fracture types (in terms of cost and impact on HRQoL and mortality), only including these locations means that the incidence of “other” fractures (i.e., non-hip, non-vertebral fractures) in the model is likely to be underestimated. This omission leads to an understatement of the burden of disease overall, and likely provides a conservative estimate of the cost-effectiveness of continued denosumab treatment; lower fracture rates lead to a smaller absolute fracture reduction for the treatment cohort, and therefore a smaller QALY gain and cost reduction from avoided fractures.

Third, this evaluation assumes that fracture rates in untreated patients remain constant over time, due to a lack of age-specific fracture data from the Taiwan real-world study. In reality, fracture incidence generally increases with age [28]. In a scenario analysis, the impact of this assumption was explored by adjusting fracture rates for age, using external data on age-specific fracture incidence in Swedish women [28]. Results of this scenario indicate that the assumption of constant fracture rates is likely to underestimate the cost-effectiveness of continued denosumab treatment.

Fourth, the index date of the Taiwan real-world study does not align exactly with the cycle length of the model. Study outcomes were recorded starting from 6 months and 45 days after the first dose of denosumab, whereas each cycle of the model begins at the time of denosumab administration. However, given the chronic nature of osteoporosis and the relatively long model cycle length, this discrepancy is unlikely to materially affect results.

Finally, as with any evaluation based on observational effectiveness data, it is impossible to rule out residual bias due to unobserved confounders. However, the design of the TWRWS (where both cohorts were selected based on treatment with denosumab, and propensity score matching was conducted using a substantial number of covariates) is likely to minimize the risk of confounding. Information on patients’ smoking and alcohol use, body mass index (BMI), and bone mineral density (BMD) were not available from Taiwan’s NHIRD, and therefore could not be included as covariates in the propensity score matching process. However, the prevalence of smoking and alcohol use is less than 5% among the elderly in Taiwan [35], and a subset analysis using records from the Chang Gung Research Database [36] showed that BMI and BMD were balanced between cohorts at baseline. Therefore, it is unlikely that these unobserved variables were associated with a substantial confounding effect.

Despite these limitations, this study provides clear evidence that continued treatment with denosumab is cost-effective compared with discontinuation after one dose, from the perspective of Taiwan’s National Health Insurance system, at a cost per QALY threshold equivalent to GPD per capita. Probabilistic sensitivity analysis and scenario analyses highlight that model conclusions are generally robust to uncertainty. Further research in this area is required, both in providing suitable Taiwan-specific data for future economic evaluations of osteoporosis treatments and in providing real-world evidence of denosumab’s effectiveness in other settings.

## Conclusion

Results of this cost-effectiveness analysis indicate that continued treatment with denosumab for up to 5 years is highly cost-effective compared with discontinuation of denosumab after one dose in a real-world setting, from the perspective of Taiwan’s healthcare system, using a willingness-to-pay threshold equivalent to national GDP per capita.

## Data Availability

Not applicable.
